# Lymphoma of the central nervous system originating from the septum pellucidum region: Two case reports with literature review

**DOI:** 10.1097/MD.0000000000035954

**Published:** 2023-11-17

**Authors:** Dawei Chen, Xu Yan, Liuzhe Lu, Kun Xue, Xuechao Dong

**Affiliations:** a Department of Neurosurgery, The First Hospital of Jilin University, Changchun, Jilin, China; b Department of Pathology, The First Hospital of Jilin University, Changchun, Jilin, China.

**Keywords:** imaging, intraventricular, primary central nervous system lymphoma, septum pellucidum, surgery

## Abstract

**Rationale:**

Non-Hodgkin lymphoma affecting the brain, eyes, and cerebrospinal fluid without systemic spread is known as primary central nervous system lymphoma (PCNSL). While intracerebroventricular PCNSL is commonly found in the lateral ventricles and the third and fourth ventricles, the occurrence of PCNSL originating from the septum pellucidum is extremely rare.

**Patient Concerns:**

Two patients presented with recent memory loss and high cranial pressure.

**Diagnoses:**

Magnetic resonance imaging revealed a clear enhancing lesion in the septum pellucidum region. Pathological examination confirmed that both cases were primary large B-cell lymphoma GCB (germinal center B-cell-like) subtypes located in an “immune-privileged” area.

**Interventions:**

Both patients underwent total tumor resection, and the procedures were successfully completed without surgical complications.

**Outcomes:**

Over a 1-year period, treatment included four cycles of high-dose methotrexate combined with temozolomide. During the follow-up period (19–23 months), no recurrence of the lymphoma was observed.

**Lessons:**

In cases of PCNSL in the septum pellucidum, it is crucial to consider it as a potential differential diagnosis for intraventricular tumors. Surgical interventions should focus on maximizing tumor resection while ensuring the protection of critical structures like the fornix and peripheral neural components. The role of surgery compared to biopsy, as well as the long-term complications, necessitates extended follow-up. Additionally, an individualized treatment approach, considering factors such as age, Karnofsky performance score, and organ function assessment, can lead to positive outcomes.

## 1. Introduction

An aggressive non-Hodgkin lymphoma that affects only the central nervous system, such as the brain, eyes, and cerebrospinal fluid, is termed primary central nervous system lymphoma (PCNSL), which accounts for 4% to 6% of all extranodal lymphomas, as well as for 4% of newly diagnosed malignant brain tumors.^[1,2]^ In PCNSL, patients exhibit focal neurologic deficits, mental status and behavioral changes, increased intracranial pressure, seizures, and other neurologic signs over a period of weeks, depending on the site of CNS involvement.^[3]^ Contrast-enhanced brain magnetic resonance imaging (MRI) is recommended for diagnosis of this condition.^[4]^ Lesions usually appear as homogenously enhanced masses on imaging. Stereotactic biopsy is commonly performed to obtain biopsy material for histological analysis, which is the gold standard for the diagnosis of PCNSL.^[5]^ Understanding of the molecular mechanism underlying the pathogenesis of diffuse large B-cell lymphoma (DLBCL) has been improved through recent advances in molecular genetics. Several pathogenetic mechanisms have been shown to be involved in PCNSL, namely mutations in specific genes (e.g., *TP53, PIM1, ATM*, and*MYD88*) as well as dysregulation in the nuclear factor kappa B (NF-kB), JAK/STAT, Toll-like receptor and B-cell receptor signaling pathways.^[6]^

Males are more likely to be diagnosed with PCNSL, and the median age at diagnosis is 60 years.^[7]^ Normally, PCNSL starts during the sixth or seventh decade of life, but it can begin earlier in immunocompromised patients.^[8]^ DLBCL accounts for about 90% of PCNSL cases, while T-cell lymphomas, poorly characterized low-grade lymphomas, and Burkitt lymphomas make up the remainder of cases.^[9]^ Immunocompetent and immunocompromised patients can be affected by PCNSL. Congenital and acquired immunosuppression are risk factors for PCNSL. In addition to immunodeficiency, other risk factors are associated with PCNSL that have not been well characterized. Immunocompetent versus immunodeficient individuals exhibit different patterns of PCNSL.^[10,11]^ It has been reported recently that the PCNSL incidence has steadily increased, especially in elderly patients over 65 years of age, who represent the majority of immunocompetent PCNSL cases.^[12,13]^ Moreover, PCNSL was identified as an independent poor prognostic factor based on age. Furthermore, elderly patients are more likely to suffer from iatrogenic toxicity than younger ones; therefore, elderly patients are a unique and vulnerable group of patients in need of treatment.^[14]^ A patient over 60 years of age is still at risk of poor outcomes, with 1-year progression-free survival rates reported to be 40% and median overall survival (OS) in the range of 8 to 43 months in elderly patients receiving multi-drug regimens, which included high-dose methotrexate (HD-MTX).^[15–21]^

In immunocompetent individuals, PCNSL typically presents as a solitary, uniformly enhanced mass, although multiple lesions are seen in 20% to 40% of cases.^[22]^ In patients with PCNSL, the white matter of the brain around the supratentorial midline is usually affected, including the thalamus/basal ganglia area, corpus callosum, paraventricular, cerebellum, brainstem and spinal cord. In most cases, lesions are located near either the ependyma or the pia,^[23,24]^ and periventricular white matter involvement is common.^[25]^ Intracerebroventricular PCNSL is mainly located in the lateral ventricles and the third and fourth ventricles.^[26,27]^ In 2013, Cheatle et al^[28]^ reported a case of PCNSL originating from the septum pellucidum in a young woman. Isolated PCNSL in the septum pellucidum has been rarely reported in immunocompetent elderly patients. In the present study, we describe 2 cases of intraventricular PCNSL in immunocompetent elderly patients.

## 2. Case presentation

### 2.1. Case report 1

**Figure 1.**
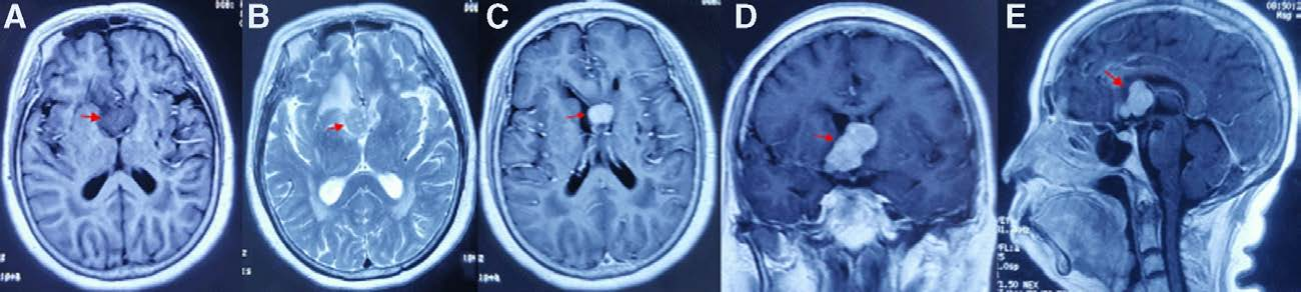
MRI T1WI image showed low signal mass, and T2WI showed mass with isosignal (A and B), and clearly nodularly enhanced lesion (C–E). MRI = magnetic resonance imaging.

A 65-year-old woman presented with a 1-month history of memory loss. She had no special history, personal history, or family history. She underwent a thorough clinical examination. Her preoperative Karnofsky performance score (KPS) was 70. On fundoscopy, no changes were observed in the optic disk or retina, and a slit-lamp examination revealed no abnormal findings. The patient showed significant recent memory loss on physical examination of cortical function (inability to recall recent breakfast and Chinese food). The remainder of the physical examination findings were negative. The results of a routine hematological examination were normal. All pituitary hormone levels were normal. Tumor marker levels remained within normal laboratory ranges. Immunological examination for HIV was negative. No suspicious lesions were found on chest/abdomen/pelvic enhanced computed tomography (CT) scans. A 3.4 × 2.6 × 2.7 cm lesion with a low signal in T1-weighted imaging (T1WI) and isosignal in T2WI was seen in the septum pellucidum region on head MRI, and the lesion was significantly enhanced after enhancement (Fig. 1A–E). Magnetic resonance spectroscopy (MRS) revealed a heightened Cho peak and a weakened N-acetylaspartate peak and significantly high lipid peak. Spinal cord enhanced MRI results were normal. The patient was placed under general anesthesia for examination by MRI-based neuronavigation. Surgery was performed through the right frontal sulcus. During the operation, the tumor was found to originate from the septum pellucidum. Macroscopically, the grayish-white tumor had a poor blood supply, a hard texture and clear boundaries with the surrounding brain tissues. The anterior inferior border reached the suprasellar level, and the posterior inferior border was at the level of the interventricular foramen anterior to the 3 ventricles.

**Figure 2.**
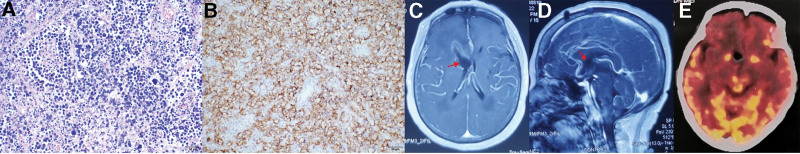
(A) The tumor cells showed diffuse infiltrative changes, with large deeply stained nuclei, little cytoplasm, tight arrangement, and rich content of reticulated fibers (hematoxylin and eosin [HE]; scale bar, 200×). (B) Tumor cells were CD20 + (scale bar, 200×). Postoperative enhanced MRI showed tumor disappearance (C and D). No abnormal hypermetabolic uptake was seen at the 23-mo postoperative follow-up on PET/CT imaging (E). MRI = magnetic resonance imaging, PET/CT = positron emission tomography/computed tomography.

An operation under a microscope was performed to remove the tumor entirely. The pathological diagnosis was PCNSL arising in immune-privileged sites, DLBCL-GCB type (Fig. 2A and B). *PIM1* and *TP53* gene mutations were found (mutation abundance 69.6%). The postoperative KPS score was 90. No complications occurred in the postoperative period. Postoperative enhanced MRI revealed disappearance of the tumor (Fig. 2C and D). Four cycles of HD-MTX (3 g/m^2^) combined with temozolomide (TMZ, 150 mg/m^2^, for 5 days every 28 days) were given after 2 weeks for 1 year. No radiotherapy was given. At the 23-month follow-up, no evidence of malignancy was found, and positron emission tomography (PET)/CT did not reveal hypermetabolic areas (Fig. 2E).

### 2.2. Case report 2

**Figure 3.**
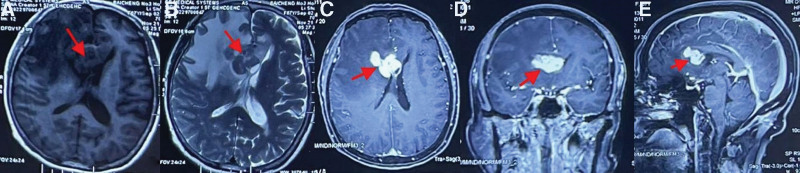
(A and B) MRI showed hypointense lesion on T1WI and T2WI; (C–E) markedly enhanced nodules. MRI = magnetic resonance imaging.

A 67-year-old woman presented with complaints of headaches and memory problems persisting for 2 months. The patient had no personal or family history of similar lesions. A detailed examination of the patient was performed before the operation. Her preoperative KPS score was 60. The results of customary slit lamp and fundus examinations were normal. The patient experienced a headache during the examination, followed by nausea and vomiting. The main symptoms also included memory loss. The remainder of the physical examination results were negative. There were no abnormal findings on routine hematological examinations, including for tumor markers. HIV testing was negative. No suspicious lesions were found on CT-enhanced scans of the chest/abdomen/pelvis. A 3.8 × 2.5 × 1.7 cm lesion with low signal on T1WI and T2WI was seen in the septum pellucidum on head MRI, and a large edematous band was seen in the right frontal lobe, which was significantly enhanced after enhancement (Fig. 3A–E). MRS revealed a heightened Cho peak, a weakened N-acetylaspartate peak and a remarkably high lipid peak. The results of enhanced MRI of the spinal cord were normal. Surgery was performed under general anesthesia with the use of neuronavigation, and a right frontal sulcus approach was used. Intraoperatively, the tumor was seen to originate from the septum pellucidum. The tumor was hard and grayish-white with a medium blood supply. Mild adhesions were found between the mass and the corpus callosum and ventricular wall.

**Figure 4.**
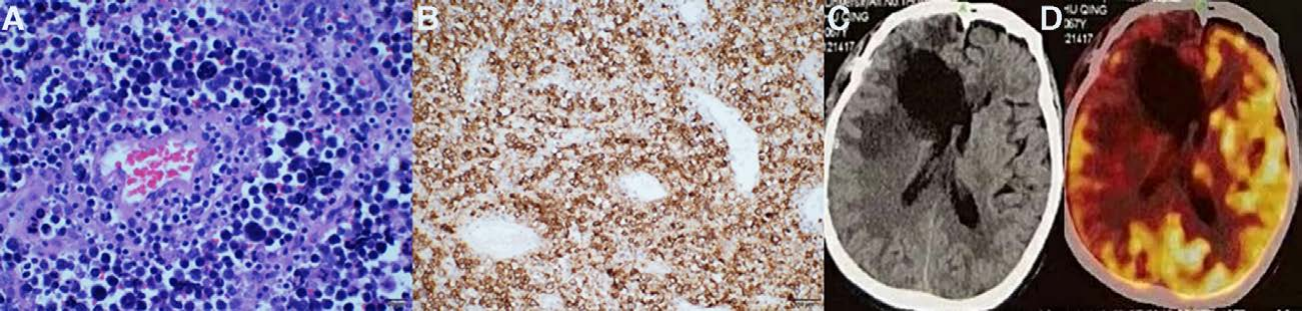
(A) The tumor cells were arranged in a cuff-like manner centered on blood vessels (HE; scale bar, 400×). (B) Tumor cells were CD79a + (scale bar, 200×). (C) Postoperative CT showed tumor disappearance. (D) No abnormal hypermetabolic uptake was seen on PET/CT at 19 mo postoperatively. PET/CT = positron emission tomography/computed tomography.

Gross total resection of the tumor was achieved. The pathological diagnosis was PCNSL arising in immune-privileged sites, DLBCL-GCB type (Fig. 4A and B) and MYD88 gene mutation (+). The postoperative KPS was 80. No complications occurred. Head CT was reviewed after the operation and proved tumor disappearance (Fig. 4C). HD-MTX (3 g/m^2^) for 4 cycles + TMZ (150 mg/m^2^, for 5 days every 28 days) was given after 3 weeks for 1 year. No radiotherapy was used. After 19 months of follow-up, there was no evidence of malignancy, and no hypermetabolic foci were detected by positron emission tomography/computed tomography (Fig. 4D).

## 3. Literature review

Located between the rostrum, genu, and anterior portion of the corpus callosum body, the septum pellucidum is a thin, transparent membrane.^[29]^ Primary tumors of the septum pellucidum are rare, accounting for approximately 0.5% of intracranial tumors.^[30]^ In the early stages of the disease, memory loss and mental disturbance are common symptoms and may be caused by destruction of memory-related structures such as the fornix.^[31]^ The cognitive symptoms of PCNSL are a crucial aspect of the diagnosis process. Cognitive assessment may sometimes be useful in distinguishing PCNSL from other nonneoplastic disorders. PCNSL is associated with more severe memory impairment than other rapidly progressive dementias, whereas neurological symptoms such as myoclonus and Parkinsonism are less common in PCNSL.^[32]^ In PCNSL, as the tumor grows, it obstructs the interventricular foramen causing symptoms of hydrocephalus and cranial hypertension.

On imaging, tumors in the septum pellucidum may have one of the following presentations: a tumor is located on one side or occupies the entire septum pellucidum, with clear borders and rare peritumoral edema; a tumor is at an acute angle to the septum pellucidum when it is small and at an obtuse angle when it is large; the septum pellucidum is deviated and a tumor is growing around the Monro foramen area or towards the third ventricle, resulting in unilateral or bilateral ventricular dilatation; or a tumor is growing into other parts of the ventricles and causing compressive edema on the surrounding brain parenchyma. However, PCNSL in the septum pellucidum has unique MRI characteristics of low or equal signal on T1WI, equal or high signal on T2WI, obvious uniform enhancement after enhancement, a high signal in diffusion-weighted imaging, a low signal in the ADC map, a lack of intra-tumor veins and neovascularization in susceptibility weighted imaging, an elevated lipid peak and elevated Cho/N-acetylaspartate ratio on MRS, and high^[18]^ F-fluoro-deoxy-glucose (FDG) uptake on positron emission tomography/computed tomography.^[33–37]^ The MRI-based differential diagnoses of PCNSL in the septum pellucidum region and other CNS tumors in this region are presented in Supplementary table, http://links.lww.com/MD/K693.

PCNSL is 95% activated B-cell-like DLBCL, with a few GCB subtypes.^[38]^ The non-GCB subtype is more likely to occur around the lateral ventricles, and the GCB subtype is more likely to occur in the midline/center and has a better prognosis.^[39]^ Genetic testing usually reveals mutations in *MYD88, CD79B, TP53, CCND3, BTG2, PIM1* and other genes.^[40]^ In the present report, both patients presented with memory loss and high cranial pressure, and the MRI and pathological manifestations were consistent with the features reported in the literature. Genetic detection of *TP53, PIM1* and *MYD88* mutations then further clarified the diagnosis.

## 4. Discussion and conclusions

Clinical diagnosis of PCNSL is based on relevant clinical features, radiological findings, and tissue biopsy results. The symptoms of PCNSL are nonspecific, but cognitive decline and gait disturbance are the most common presentations,^[41]^ as seen in the initial presentations of our patients. More than half of patients with PCNSL suffer from disturbances in the cerebral hemispheres and basal ganglia.^[42,43]^ T1WI of lymphoma lesions in the CNS commonly show hypointense lesions, and these lesions are iso- to hyperintense on T2WI. Lesions in the brain are generally well-defined and well-separated from the surrounding brain parenchyma, with minimal peritumoral edema.^[44]^ Corticosteroid are believed to greatly influence imaging findings if they lead to shrinkage or disappearance of a tumor, thereby limiting the imaging findings and confounding biopsy results. Thus, imaging and biopsy collection must be performed before steroids are administered. In some cases of PCNSL, a lesion with a diffuse border can be seen, even without the formation of a distinct mass, mimicking a malignant glioma. Histopathology in such cases revealed that the tumor cells invade the neural parenchyma in small clusters or single cells with angiocentric accentuation.^[45]^ Chest, abdomen, and pelvic screening examinations are also needed to confirm that secondary lymphoma is not present.^[8,46,47]^ In cases of suspected PCNSL, further diagnostic evaluation is essential, including slit-lamp evaluation to detect involvement of the intraocular tissues, which reportedly occurs in about 20% of PCNSL cases.^[48]^

Previous research has reported a number of specific characteristics of PCNSL, including infiltrative growth, location in deep or functional areas of the brain, a propensity for cerebrospinal fluid and meningeal dissemination, and sensitivity to radiotherapy and chemotherapy. Many previous studies concluded that surgical treatment is associated with a high risk of complications, minimal clinical benefit, and delayed chemotherapy, and thus, the current literature mostly recommends surgery only in cases with brain herniation causing elevated intracranial pressure.^[4,49–53]^ In recent years, numerous studies have reassessed the role of surgery in PCNSL treatment,^[3,54–57]^ arguing that the earlier studies were small-sample, single-center, retrospective stud done in the absence of standard chemotherapy. Cloney et al concluded that surgical resection of PCNSL was safe for selected patients and had complication rates comparable to other intracranial tumors, with a meta-analysis showing no difference in complications between patients who underwent surgical resection and those who underwent biopsy.^[58]^ However, meta-analysis of more than 1000 patients with PCNSL showed a significantly lower risk of death and disease progression in patients who underwent resection compared to those who underwent biopsy only.^[59]^

The application of modern precision microsurgery techniques and equipment (including but not limited to multimodal MRI, neuronavigation, intraoperative nuclear magnetic, neuroendoscopy, neuroelectrophysiology, etc) allows for total tumor resection with maximum protection of neurological function. Therefore, at this time, the advantages of surgery have become progressively more pronounced. Surgery can quickly relieve tumor compression, improve symptoms, clarify pathological diagnosis and shorten the course of chemotherapy. Gross resection of the complete tumor via surgery is an independent prognostic factor for long-term survival.^[54]^ In several studies, surgical excision combined with chemotherapy led to better outcomes than chemotherapy alone, suggesting that multimodal treatment may be more beneficial. Notably, the extent of tumor resection was positively correlated with survival, with a median survival superior to stereotactic brain biopsy and postoperative complications similar to those observed for other intracranial tumors.^[54,60–64]^ In terms of the specific extent of excision, more extensive resection was associated with better survival.^[65]^ Rae et al^[61]^ and Ouyang et al^[66]^ reported that craniotomy improves the survival rate of PCNSL patients more than biopsy. A single-center study of 167 PCNSL cases showed that the benefit was more pronounced in those who underwent complete tumor resection.^[67]^ A study of 4812 PCNSL cases from the SEER database in the United States showed that complete or partial resection of the tumor improved the prognosis and survival time of patients.^[68]^ Mario et al^[69]^ reported good results for neuroendoscopic resection of intracerebroventricular PCNSL treatment. Patients with PCNSL who received adjuvant radiation therapy (RT) after surgery had a significant survival advantage, according to Kinslow et al.^[70]^ Thus, numerous variables affect the decision regarding whether surgery can benefit a patient with PCNSL. It may be reasonable to consider surgery for patients with a large primary lesion causing a prominent tumor burden in order to improve the patient quality of life.^[54]^ Several factors must be considered in the clinical decision to pursue a debulking surgical procedure, such as the patient age, performance status, and deep brain structure involvement.^[71]^ Surgical intervention may not be an option when tumors are diffuse and affect multiple neurologic structures.

For PCNSL in the hyaloid septal area, preoperative assessment should be adequate, and the frontal cortex, sulcus, and corpus callosum-callosal interspace approaches should be taken according to the location of the tumor and the experience of the operator. Intraoperative accurate judgement and protection of the fornix and peripheral neural structures are the difficulty and key to success of the surgery. We propose that the following points should be given attention during surgery: the fornix embedded in the corpus callosum is a possible site of fornix injury during surgery; the depth of downward separation should be carefully observed when separating the septum lucidum/interseptal space; the fornix should be separated bilaterally through the posterior inferior part of the septum lucidum; and when separating the septum lucidum and the fornix posteriorly, care must be taken to avoid injury of the fornix columns anteriorly to the anterior part of the interventricular foramen. For the 2 patients described in this report, the morphology and position of the fornix and its surrounding structures were altered by the tumor, and the sulcus-lateral ventricle-interventricular foramen approach was used after adequate preoperative assessment by MRI in coronal and sagittal positions, which proved to be suitable for these cases. The operating space and surgical freedom within the lateral ventricle were good. The tumor was clearly delineated from the ventricular wall and mildly adhered to the corpus callosum, and the interventricular foramen was not completely obstructed. The tumor was isolated and completely resected in accordance with the authors’ experience as described above, which effectively preserved the fornix and the peripheral neurovascular structures and avoided the risk of hydrocephalus and metastasis of tumor after implantation of the palliative V-P shunt. Significant improvement in the patients’ symptoms was observed postoperatively without complications.

There is no standard drug treatment for PCNSL, and HD-MTX–based combination chemotherapy is currently preferred. When combined with TMZ, significantly improved progression-free survival and OS are observed. TMZ readily crosses the blood–brain barrier.^[11,72]^ Both cases in this report were treated with HD-MTX combined with TMZ (high economic benefit, good blood–brain barrier permeability, low neurotoxicity), and no RT was administered.No tumor recurrence was seen at 19–23 months of follow-up. In the future, with the use of new targeted inhibitors (targeting Bruton tyrosine kinase [BTK], mammalian target of rapamyocin [mTOR], phosphoinositide 3-kinase [PI3K], and exportin 1), immune checkpoint inhibitors (targeting programmed death receptor-1), immunomodulators and extensive research on chimeric antigen receptor (CAR)-T cell therapy, the prognosis of patients will surely be further improved.^[73]^

Overall, primary CNS lymphomas are underdiagnosed in immunocompetent individuals due to their low incidence and nonspecific symptoms. Primary CNS lymphoma in the septum pellucidum region in the elderly with normal immune function is rare. The 2 patients in our current report were presented with memory loss and increased cranial pressure as the first symptoms, which need to be differentiated by neuroimaging from intracerebroventricular central neuroblastoma and others. The tumor was completely removed using a precise microsurgical technique, and it was clear intraoperatively that the tumor originated in the hyaline septum. The fornix column and corpus callosum were well protected during the surgery, and patients with postoperative memory loss recovered to varying degrees without cognitive or mental impairment. The postoperative treatment plan, HD-MTX combined with TMZ, which is economical, has good blood-brain barrier permeability and low neurotoxicity, has achieved good results. Memory, emotion, and mental disorders in septum pellucidum may be caused by the degeneration of the surrounding structures (such as the callosum, the fornix, and the hypothalamus). Intraoperative identification and protection of the fornix and peripheral neural structures are difficult but critical to the success of the surgery due to the disruption and displacement of normal anatomical structures by the tumor. The follow-up time for the 2 presented cases was short. Longer follow-up is needed to confirm whether surgical resection of the tumor and administration of combination treatments can improve the prognosis and survival time as compared to biopsy alone, and whether potential complications such as cognitive deficits will occur in the distant future. The results of this study are summarized in the following table.

## Acknowledgments

The authors would like to thank Medjaden Bioscience Limited (https://www.medjaden.com/show-790.html) for English language editing and review services.

## Author contributions

**Formal analysis:** Xu Yan, Kun Xue.

**Writing – original draft:** Liuzhe Lu, Xuechao Dong.

**Writing – review & editing:** Dawei Chen.

## Supplementary Material


