# Supplementary material for: Lymphoma of the central nervous system originating from the septum pellucidum region: Two case reports with literature review
Source: Medicine (Baltimore). 2023 Nov 17;102(46):e35954. doi: 10.1097/MD.0000000000035954 (PMC10659664; doi:10.1097/MD.0000000000035954)
Supplement: Supplementary file 1 [file medi-102-e35954-s001.docx]

Supplementary Table. MRI manifestations of CNS tumors in the septum pellucidum

| Tumors | Age at onset | (TIW1) (TIW2) | Enhancement | Calcification | Pattern |
| --- | --- | --- | --- | --- | --- |
| PCNSL | Middle and elderly | (E/L) (E/H) | Marked homogeneous | No | No |
| CNC | Young and middle | (E/L) (E/H) | Heterogeneous | Yes | Cystic or solid-cystic |
| GG | Children and youth | (E/L) (E/H) | Ring | Yes | Cystic or solid-cystic |
| Subependymoma | Middle and elderly | (E/L) (H) | No | Punctate | Solid-cystic |
| Ependymoma | Adolescence | (L) (H) | Heterogeneous | Yes | Solid-cystic |
| Hemangioma Cavernosum | Middle-aged | (L) (H) | No/mild | Yes | Yes |
| Germ cell tumor | Childood and adolescence | (L) (H) | Marked homogeneous | Yes | No |
| Colloid cyst | Middle-aged | (H) (L/H) | No | No | Yes |
| Astrocytoma | Middle and elderly | (E/L) (E/H) | Ring or butterfly-shaped | Yes | Yes |
| SGCA | Childood and adolescence | (E/L) (E/H) | Heterogeneous | Yes | Yes |
| Lipoma | Adolescence | (H) (H) | No | No | No |

PCNSL=primary central nervoussystem lymphoma; CNC=Central neurocytoma; GG=ganglioglioma; SGCA=subependymalgiantcella; E=equal signal; L=low signal; H=high signal.
